# MSI1-FTHL17C-iron circuit couples metabolic and epigenetic control of pluripotency in mouse embryonic stem cells

**DOI:** 10.1186/s13619-026-00288-8

**Published:** 2026-05-06

**Authors:** Qianyan Li, Yi Li, Jiazhen Han, Liming Cheng, Gufa Lin, Youwei Chen

**Affiliations:** 1 Key Laboratory of Spine and Spinal Cord Injury Repair and Regeneration of Ministry of Education, Orthopaedic Department of Tongji Hospital, School of Life Sciences and Technology, Tongji University, Shanghai, China; 2 Clinical Center for Brain and Spinal Cord Research, Medical School, Tongji University, Shanghai, China

**Keywords:** MSI1, Fthl17c, Pluripotency, Iron metabolism, TET enzyme, DNA demethylation, MRNA stability, Embryonic stem cells

## Abstract

Maintenance of pluripotency in embryonic stem cells (ESCs) requires coordinated integration of transcriptional, metabolic, and epigenetic programs. Here, we identify a post-transcriptional regulatory axis linking the RNA-binding protein Musashi-1 (MSI1) to iron dependent DNA demethylation via the ferritin like gene *Fthl17c*. Genetic ablation of MSI1 and its short isoform MSI1-C in mESCs induced spontaneous differentiation and was accompanied by downregulation of *Fthl17* family genes. Among these, *Fthl17c* was directly bound and stabilized by MSI1, and its depletion reduced intracellular ferrous iron (Fe^2^⁺), impaired ten eleven translocation (TET) enzyme activity, and increased global 5-methylcytosine (5mC) levels. Restoration of *Fthl17c* expression rescued TET activity, reduced DNA methylation, and reinstated pluripotency associated gene expression, whereas extracellular Fe^2^⁺ supplementation alone was insufficient. In contrast, vitamin C, which preserves redox active Fe^2^⁺, effectively restored DNA demethylation, highlighting the requirement for bioavailable iron in TET mediated epigenetic regulation. Biochemical and imaging analyses further revealed that FTHL17C interacts with TET1 in the nucleus, supporting a role in facilitating iron dependent catalysis. Together, these findings define an MSI1-FTHL17C-Fe^2^⁺-TET axis that integrates post transcriptional control of iron homeostasis with epigenetic remodeling to preserve the pluripotent and plastic state of embryonic stem cells.

**Supplementary Information:**

The online version contains supplementary material available at 10.1186/s13619-026-00288-8.

## Background

Embryonic stem cells (ESCs) are pluripotent cells derived from the early mammalian embryo that can self-renew indefinitely while retaining the capacity to generate all somatic lineages. These properties make ESCs central to developmental biology and regenerative medicine (Lee et al. 2017; Selwood And Johnson 2006; Weinberger et al. 2016; Yanagida et al. 2021). ESC identity is maintained by a tightly regulated gene expression program centered on the transcription factors POU5F1, SOX2, and NANOG, which form interconnected feedback loops to sustain pluripotency and self-renewal (Boyer et al. 2005; Heurtier et al. 2019; Jaenisch And Young 2008; Navarro et al. 2012). Additional transcriptional regulators and extrinsic signaling pathways, including LIF/STAT3 in mouse ESCs and Activin/Nodal signaling in human ESCs, further stabilize the pluripotent state (Adachi et al. 2018; Hall et al. 2009; Loh And Lim 2011; Vallier et al. 2005; Wang et al. 2012; Weinberger et al. 2016).

Beyond transcriptional control, post transcriptional regulation and epigenetic mechanisms are essential for maintaining ESC pluripotency (Boland et al. 2014; Chen And Hu 2017; Gokbuget And Blelloch 2019). RNA-binding proteins (RBPs) regulate mRNA stability and translation and can profoundly influence pluripotency and reprogramming efficiency (Chen And Hu 2017; Ye And Blelloch 2014; Tahmasebi et al. 2014). Among these, Musashi-1 (MSI1) is a conserved RBP required for stem cell maintenance and has been shown to be indispensable for naïve pluripotency in mouse ESCs; deletion of MSI1 and its short isoform (MSI1-C) leads to early developmental arrest and loss of pluripotency (Chen et al. 2023; Sakakibara et al. 2002). In parallel, ESCs exhibit a unique epigenomic landscape characterized by open chromatin and globally low DNA methylation (Kobayashi And Kikyo 2015; Marks et al. 2012; Shukla et al. 2020; Skelly et al. 2020; Smith et al. 2012). This state is actively maintained by TET family DNA dioxygenases, which generate 5-hydroxymethylcytosine (5hmC) and restrain premature lineage priming (Hon et al. 2014; Huang et al. 2014; Ito et al. 2010; Koh et al. 2011).

Evidence indicates that cellular metabolism directly influences the epigenetic landscape of pluripotent stem cells. Metabolic intermediates generated from central carbon metabolism serve as essential cofactors or substrates for chromatin-modifying enzymes, thereby coupling metabolic state to epigenetic regulation. For example, α-ketoglutarate derived from the tricarboxylic acid (TCA) cycle supports histone demethylation and maintains pluripotency (Carey et al. 2015), whereas metabolic rewiring affecting acetyl-CoA or S-adenosylmethionine levels can alter histone acetylation and DNA methylation states (Moussaieff et al. 2015; Sperber et al. 2015). These findings highlight metabolism as a key regulator of chromatin dynamics in ESCs.

TET enzymes require ferrous iron (Fe^2^⁺) as an essential cofactor, linking DNA demethylation to intracellular iron availability and redox state (An et al. 2017; Tahiliani et al. 2009; Zhao et al. 2014). Vitamin C enhances TET activity by maintaining Fe^2^⁺ in a reduced state and induces global increases in 5hmC in ESCs (Blaschke et al. 2013). Despite this dependence, the mechanisms that ensure nuclear Fe^2^⁺ availability in ESCs remain largely unknown. Iron metabolism is primarily regulated by cytoplasmic ferritins and membrane transporters, whereas nuclear iron handling is poorly characterized (Ru et al. 2024; Suzuki et al. 2023). One potential regulator is the ferritin like protein FTHL17, identified among candidate reprogramming factors and specifically expressed in ESCs and germ cells (Kobayashi et al. 2010; Takahashi And Yamanaka 2006). Although FTHL17 proteins resemble ferritin heavy chains, they lack ferroxidase activity and exhibit partial nuclear localization, suggesting a noncanonical role in iron regulation (Ruzzenenti et al. 2015). Here, we demonstrate that MSI1 maintains ESC pluripotency by regulating *Fthl17* expression and nuclear iron homeostasis, thereby sustaining TET dependent DNA demethylation and the pluripotent epigenetic state.

## Results

### Transcriptomic network analysis identifies Fthl17 family genes as downstream targets of MSI1/MSI1-C

**Fig. 1:**
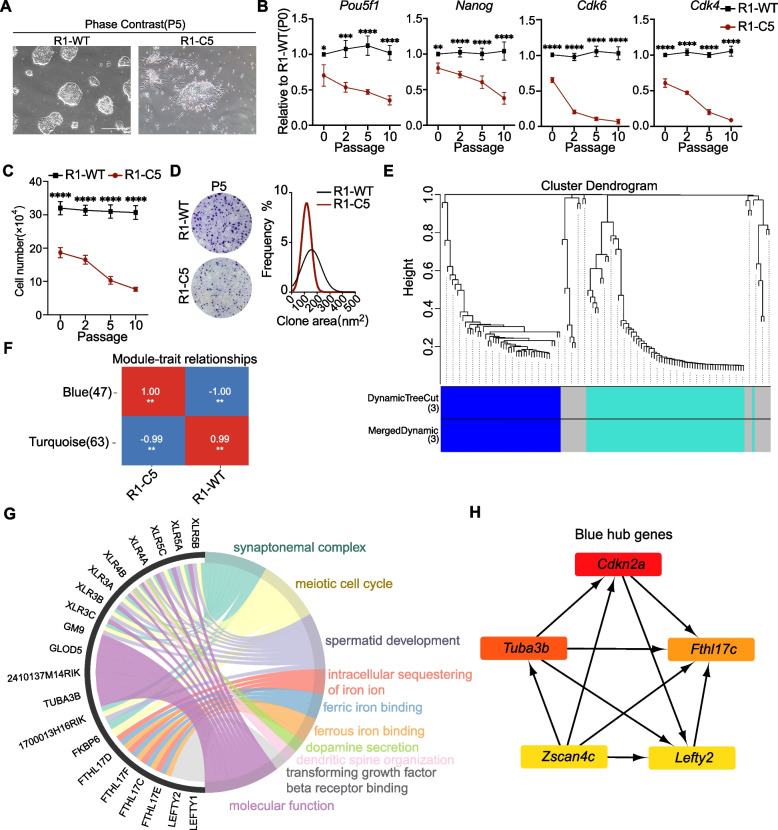
Transcriptomic network analysis reveals iron-regulatory genes as downstream targets of MSI1 in mouse ESCs. **A** Phase-contrast images showing the morphology of wild-type R1 mouse embryonic stem cells (ESCs) and MSI1/MSI1-C double knockout cells (R1-C5). R1-C5 cells exhibit spontaneous differentiation during serial passage. Cells were cultured on gelatin-coated plates. Scale bar, 100 µm. **B** RT-qPCR analysis of the expression of pluripotency genes (*Pou5f1* and *Nanog*) and cell cycle related genes (*Cdk6* and *Cdk4*) in R1-WT and R1-C5 cells at passages P0, P2, P5, and P10. **C** Cell numbers measured 72 h after seeding 50,000 cells. **D** Representative images of P5 R1-WT and R1-C5 colonies stained with crystal violet and the corresponding frequency distribution of colony sizes. **E** Weighted gene co-expression network analysis (WGCNA) of transcriptomes from R1-WT and R1-C5 cells identified major co-expression modules. **F** Module-trait correlation analysis highlights the blue module as positively associated with R1-C5 (differentiated) and negatively correlated with R1-WT (pluripotent) cells. **G** Gene Ontology (GO) enrichment analysis of genes in the blue module shows significant enrichment in gametogenesis-related pathways and iron ion metabolism (e.g., intracellular iron sequestration and ferrous ion binding). **H** Hub gene network of the blue module, with *Fthl17c* identified as one of the central nodes. All data represent mean ± SD from three independent experiments. Statistical significance was assessed using unpaired two-tailed t tests. *p < 0.05, **p < 0.01, ***p < 0.001, ****p < 0.0001; ns, not significant

In our previous study, we generated a monoclonal MSI1/MSI1-C double knockout mouse embryonic stem cell line (R1-C5) using CRISPR/Cas9 (Fig. S1A-B) (Chen et al. 2023). The initially identified R1-C5 cells were defined as passage 0 (P0). Upon serial passaging, R1-C5 cells progressively lost pluripotency. Notably, at five passages (P5), R1-C5 exhibited differentiated colony morphology and reduced expression of pluripotency markers (Fig. 1A, S1C).

RT-qPCR analysis further revealed passage dependent downregulation of the pluripotency genes *Pou5f1* and *Nanog*, together with decreased expression of cell cycle regulators *Cdk4* and *Cdk6* (Fig. 1B). Consistent with these observations, proliferation assays showed that R1-C5 cells displayed progressively impaired growth compared with R1-WT cells. When 50,000 cells were seeded and cultured for 72 h, the number of R1-C5 cells declined markedly with passage number (P0-P10), whereas R1-WT cells maintained stable proliferative capacity (Fig. 1C). In addition, crystal violet staining and colony size distribution analysis at P5 demonstrated that R1-C5 formed markedly smaller colonies than R1-WT cells, indicating reduced proliferative capacity (Fig. 1D).

To investigate transcriptional changes underlying this phenotype, we re-analyzed previously published RNA-seq data from R1-WT and R1-C5 cells using weighted gene co-expression network analysis (WGCNA). This analysis identified two major gene modules with opposing associations to cell state (Fig. 1E-F). Genes within the blue module were preferentially expressed in R1-WT cells, whereas genes in the turquoise module were enriched in R1-C5 cells and associated with differentiation related processes, including endodermal development (Fig. S1D-F). Consistently, expression of endodermal transcription factors such as *Gata4*, *Gata6*, *Foxa2*, and *Cxcr4* was significantly upregulated in R1-C5 cells, as confirmed by RT-qPCR and immunofluorescence analysis (Fig. S1G-H).

Focusing on the module downregulated upon MSI1/MSI1-C loss (Blue module), Gene Ontology (GO) enrichment analysis revealed significant enrichment in pathways related to gametogenesis and iron ion metabolism, including intracellular iron sequestration and iron binding (Fig. 1G). Network based hub gene analysis identified *Fthl17c* among the central nodes of this module, together with pluripotency associated genes *Lefty2* and *Zscan4* (Fan et al. 2013; Kim et al. 2014; Troiano et al. 2020) and cell cycle associated genes *Cdkn2a* (Fig. 1H).

*Fthl17c* belongs to the ferritin heavy chain like (*Fthl17*) gene family, whose members are specifically expressed in ESCs and lack canonical ferroxidase activity (Ruzzenenti et al. 2015). Notably, GO terms associated with *Fthl17* family genes were uniquely linked to iron binding and intracellular iron homeostasis, distinguishing them from other hub genes within the module. These findings suggest that *Fthl17* family genes act as downstream targets of MSI1/MSI1-C and may couple iron metabolism to epigenetic regulation of pluripotency.

### MSI1/MSI1-C directly binds to and stabilizes Fthl17c mRNA

To validate the biological relevance of this network based prediction, we next performed targeted functional assays to assess the role of *Fthl17* downstream of MSI1/MSI1-C.

The mouse *Fthl17* gene family comprises six highly homologous paralogs located on the X chromosome (*Fthl17a-f*). To assess the internal similarity of this family, we performed sequence identity analysis, which revealed that all members except *Fthl17a* share high coding sequence (CDS) and amino acid similarity (Fig. S2A). Phylogenetic analysis based on protein sequences further confirmed that *Fthl17a* diverges more distantly from the remaining paralogs, which cluster tightly together (Fig. S2B), suggesting potential redundancy or conserved functions within the latter group.

Expression analysis in R1-WT and R1-C5 cells revealed that *Fthl17c-f* were significantly downregulated in R1-C5, whereas *Fthl17a* and *Fthl17b* showed minimal change (Fig. S2C). In parallel, analysis of previously published MSI1 RIP-seq data demonstrated the strongest binding peak over the *Fthl17c* transcript among all family members (Fig. S2D). Taken together, these findings support the rationale for focusing on *Fthl17c* as a representative gene in subsequent functional experiments.

**Fig. 2:**
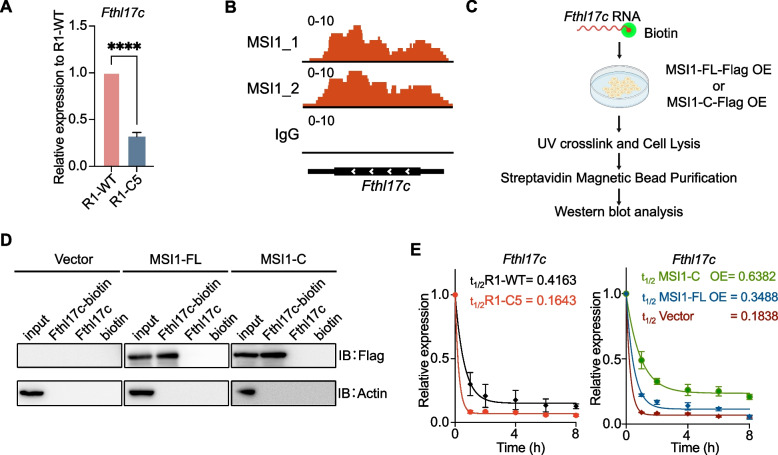
MSI1/MSI1-C directly binds to and stabilizes *Fthl17c* mRNA. **A** RT-qPCR analysis of Fthl17c mRNA levels in R1-WT and R1-C5 cells. Expression is significantly reduced in R1-C5 cells. Data represent mean ± SD from three independent experiments, unpaired two-tailed t test, ****p < 0.0001. **B** Integrative Genomics Viewer (IGV) tracks showing MSI1 RIP-seq reads mapped to the *Fthl17c* locus in R1 mouse ESCs. The x-axis denotes genomic position; the y-axis indicates read coverage. **C** Schematic of the biotin-labeled RNA capture assay with UV cross-linking used to detect RNA-protein interactions. **D** Western blot showing that both MSI1-FL and MSI1-C isoforms bind biotinylated *Fthl17c* RNA in 293FT cells, confirming direct interaction. Biotin alone was used as a negative control. **E** RNA stability analysis of *Fthl17c* mRNA in R1-WT and R1-C5 cells following actinomycin D treatment (0-8 h). MSI1-FL and MSI1-C overexpression prolongs transcript half-life in R1-C5 cells. Data are normalized to 0 h. Nolin fit curves with half-life (calculated from three independent replicates) are included. Vector overexpression served as the baseline control

We first validated the downregulation of *Fthl17c* in R1-C5 cells. RT-qPCR analysis revealed a marked downregulation of *Fthl17c* mRNA in R1-C5 cells compared with R1-WT controls (Fig. 2A). To explore whether *Fthl17c* (a representative member of the *Fthl17* family) is a direct post transcriptional target of MSI1, we examined MSI1 RIP-seq data. Integrated Genome Viewer (IGV) plots showed clear MSI1 binding peaks located within the *Fthl17c* transcripts (Fig. 2B, S2D).

To validate this interaction in living cells, we performed a biotin labeled RNA capture assay combined with UV cross linking. *Fthl17c* mRNA was transcribed in vitro, biotinylated at its 3′ end, and transfected into 293FT cells overexpressing MSI1-FL or MSI1-C. After UV cross linking to stabilize RNA-protein complexes, cells were lysed, and the biotinylated RNA-protein complexes were purified using streptavidin magnetic beads. Western blot analysis confirmed that both MSI1-FL and MSI1-C were efficiently recovered with biotinylated *Fthl17c* RNA, demonstrating a direct association between both isoforms and *Fthl17c* transcripts in vivo (Fig. 2C-D).

To test whether this interaction influences RNA stability, we measured *Fthl17c* mRNA half-life in R1-WT and R1-C5 cells after actinomycin D treatment. *Fthl17c* mRNA decayed more rapidly in R1-C5 cells than in R1-WT, whereas overexpression of MSI1-FL partially restored stability, and MSI1-C overexpression markedly prolonged the transcript half-life (Fig. 2E).

Together, these results demonstrate that *Fthl17c* is a direct downstream target of MSI1/MSI1-C and that both isoforms bind to and stabilize *Fthl17c* mRNA, thereby maintaining its expression in embryonic stem cells.

### *Fthl17c* regulates pluripotency and intracellular iron homeostasis in embryonic stem cells

To determine whether *Fthl17c* plays a functional role in maintaining mESC pluripotency, we overexpressed *Fthl17c* in the MSI1/MSI1-C deficient R1-C5 cell line and examined pluripotency associated gene expression. To determine whether the rescue effect of *Fthl17c* depends on the passage status of R1-C5 cells, we first overexpressed *Fthl17c* in R1-C5 cells at different passages (P0, P2, P5, and P10) and analyzed pluripotency marker expression 72 h after lentiviral transduction. RT-qPCR analysis showed that *Fthl17c* incrfvaeased the expression of the pluripotency genes *Pou5f1* and *Nanog* at all tested passages (Fig. S3A). However, the magnitude of rescue gradually declined as passage number increased. We then asked whether this rescue effect could be maintained after serial passaging. To this end, *Fthl17c* overexpression was established in R1-C5 cells at P5 or P10, and cells were further passaged and collected at P0, P2, P5, and P10 after lentiviral transduction. Under these conditions, restored expression of pluripotency markers remained detectable in both P5- and P10-derived R1-C5 cells after serial passaging (Fig. S3B).

**Fig. 3:**
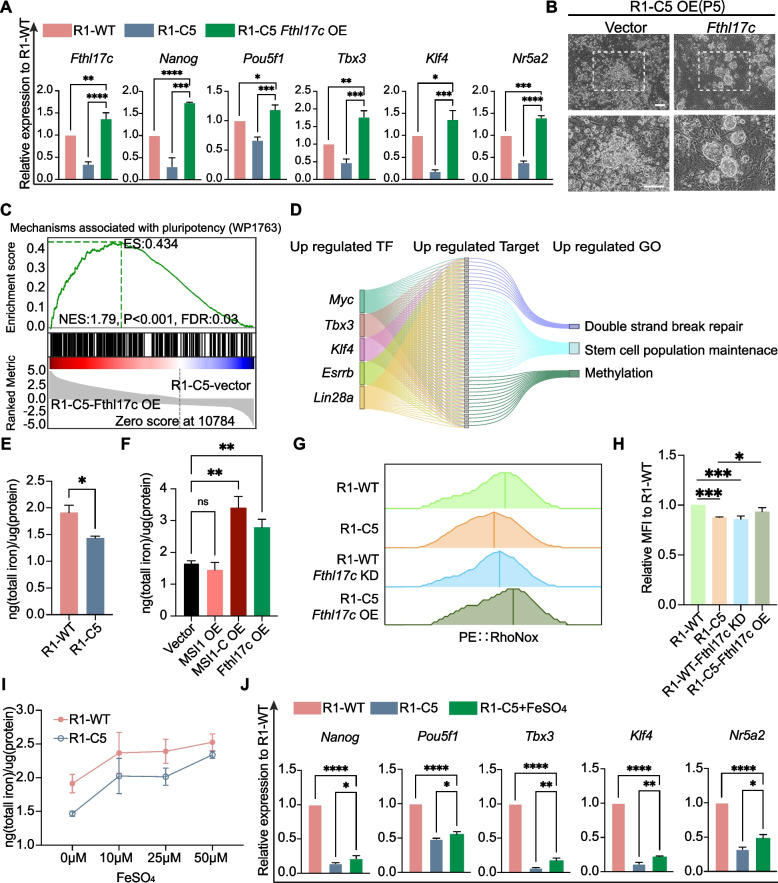
*Fthl17c* regulates pluripotency and intracellular iron homeostasis in embryonic stem cells. **A** RT-qPCR analysis of pluripotency marker genes (*Nanog, Pou5f1*, *Tbx3*, *Klf4*, *Nr5a2*) in R1-WT, R1-C5, *Fthl17c* overexpressing R1-C5. **B** Phase-contrast images showing colony morphology of ESCs and *Fthl17c* overexpressing R1-C5 cells. Scale bar, 100 µm. **C** Gene set enrichment analysis (GSEA) of differentially expressed genes following *Fthl17c* overexpression in R1-C5 cells. The enrichment plot shows significant upregulation of a pluripotency-associated gene set, with normalized enrichment score (NES), nominal *p* value, and false discovery rate (FDR) indicated. **D** Sankey diagram illustrating the relationships between enriched transcription factors and pluripotency related Gene Ontology (GO) terms identified upon *Fthl17c* overexpression. The flow width represents the number of target genes contributing to each transcription factor GO category association. **E**–**F** Quantification of total intracellular iron in R1-WT, R1-C5 (**E**), and *Msi1*, *Msi1-C* and *Fthl17c* overexpressing R1-C5 cells (**F**). **G**-**H** Flow cytometric detection (**G**) and mean fluorescence intensity (MFI) quantification (**H**) of labile Fe^2^⁺ using RhoNox-1 in R1-WT, R1-C5, *Fthl17c* knockdown R1-WT, and *Fthl17c* overexpressing R1-C5 cells. **I** Total intracellular iron content in R1-WT and R1-C5 cells treated with increasing concentrations of FeSO_4_(0-50 µM). **J** RT-qPCR analysis showing that Fe^2^⁺ supplementation fails to restore pluripotency marker expression in R1-C5 cells. All data represent mean ± SD from three independent experiments. Statistical significance was assessed using unpaired two-tailed t tests (**E**) and one-way ANOVA (**A**, **F**, **H**, **J**). *p < 0.05, **p < 0.01, ***p < 0.001, ****p < 0.0001; ns, not significant

Consistently, overexpression of *Fthl17c* significantly restored the expression of *Nanog*, *Pouf5f1*, *Tbx3*, *Klf4*, and *Nr5a2*, reaching levels comparable to or exceeding those observed in R1-WT cells (Fig. 3A). Consistently, *Fthl17c* overexpressing R1-C5 colonies displayed compact, dome shaped morphology characteristic of undifferentiated ESCs, in contrast to vector control cells (Fig. 3B). Conversely, knockdown of *Fthl17c* in R1-WT cells resulted in downregulation of pluripotency genes (*Nanog*, *Pou5f1*, *Tbx3*, *Klf4*, and *Nr5a2*) (Fig. S3C).

Gene set enrichment analysis (GSEA) showed that pluripotency associated gene signatures were significantly enriched following *Fthl17c* overexpression in R1-C5 cells (Fig. 3C). The Sankey diagram shows that transcription factor genes *Myc, Tbx3*, *Klf4*, and *Lin28a* were upregulated following *Fthl17c* overexpression, and their upregulated target gene were enriched in GO terms related to double strand break repair, stem cell population maintenance, and methylation (Fig. 3D). This indicates that *Fthl17c* overexpression restores the pluripotency regulatory network.

Given that *Fthl17c* was enriched in iron ion metabolism related GO terms, we next examined whether it affects intracellular iron homeostasis. Quantification of total cellular iron revealed a significant reduction in R1-C5 cells compared with R1-WT cells (Fig. 3E). Overexpression of MSI1-C largely restored total iron levels in R1-C5 cells, and overexpression of *Fthl17c* increased intracellular iron levels comparable to MSI1-C overexpression (Fig. 3F). These results support the idea that *Fthl17c* functions downstream of MSI1-C in regulating intracellular iron homeostasis.

We next assessed the labile ferrous iron (Fe^2^⁺) pool using the fluorescent probe RhoNox-1. Flow cytometric analysis showed that R1-C5 cells and *Fthl17c* knockdown R1-WT cells exhibited significantly reduced Fe^2^⁺ fluorescence relative to R1-WT controls. In contrast, *Fthl17c* overexpression in R1-C5 cells markedly increased intracellular Fe^2^⁺ levels (Fig. 3G-H).

To minimize potential optical bias caused by the three-dimensional morphology of ESC colonies, cells were dissociated into single cells prior to RhoNox staining. Nuclear Fe^2^⁺ levels were then quantified by measuring RhoNox fluorescence intensity normalized to Hoechst staining. Imaging analysis revealed that knockdown of *Fthl17c* in R1-WT cells significantly reduced nuclear Fe^2^⁺ signals, whereas overexpression of *Fthl17c* in R1-C5 cells markedly increased nuclear fluorescence intensity (Fig. S3D-E).

We next tested whether exogenous Fe^2^⁺ supplementation could compensate for *Fthl17c* loss. Although ferrous sulfate treatment elevated total iron levels in both R1-WT and R1-C5 cells, the iron content in R1-C5 remained lower than that in R1-WT (Fig. 3I). Moreover, RT-qPCR showed that Fe^2^⁺ supplementation failed to significantly restore the expression of pluripotency associated genes in R1-C5 cells (Fig. 3J). Consistently, ferrous sulfate increased cytoplasmic fluorescence but did not significantly elevate nuclear RhoNox signals (Fig. S3F). Quantification of isolated nuclei revealed that nuclear iron levels were significantly reduced in R1-C5 and R1-WT *Fthl17c* knockdown cells, whereas *Fthl17c* overexpression restored nuclear iron levels (Fig. S3G).

Together, these results establish *Fthl17c* as a key regulator of pluripotency and intracellular iron homeostasis in embryonic stem cells.

### *Fthl17c* deficiency reduces TET enzyme activity and increases DNA methylation levels

**Fig. 4:**
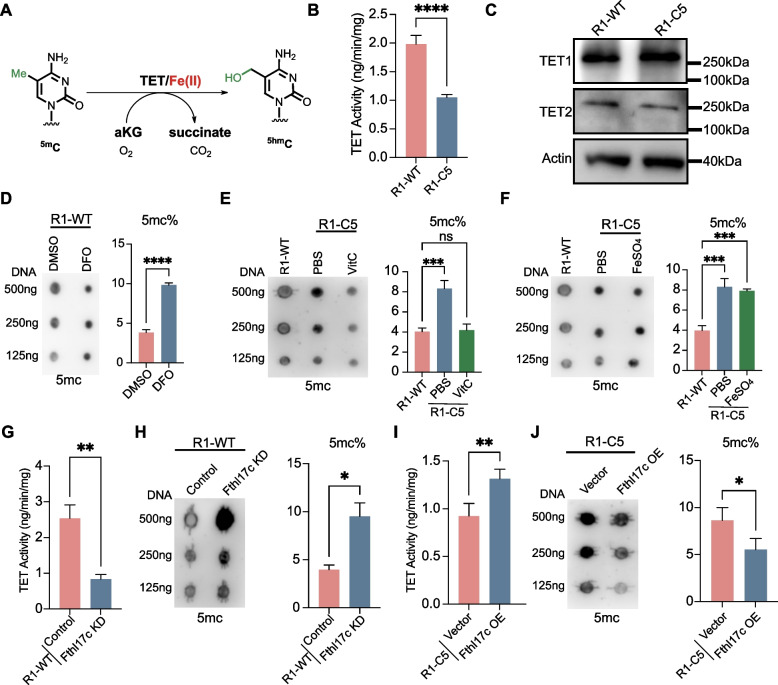
*Fthl17c* deficiency impairs TET enzyme activity and increases global DNA methylation. **A** Schematic illustrating the oxidation of 5-methylcytosine (5mC) to 5-hydroxymethylcytosine (5hmC) catalyzed by TET enzymes in a Fe^2^⁺- and α-KG-dependent reaction. **B** Quantification of total TET enzymatic activity in R1-WT and R1-C5 cells. **C** Western blot showing comparable TET1 and TET2 protein levels in R1-WT and R1-C5 cells. **D** Dot blot and 5mc ELISA analysis of 5mC levels in R1-WT cells treated with deferoxamine (DFO, 12.5 µM) or DMSO control. **E** Dot blot and 5mc ELISA analysis of 5mC levels in R1-C5 cells treated with vitamin C (L-ascorbic acid, 100 µM) or PBS. **F** Dot blot and 5mc ELISA analysis of 5mC levels in R1-C5 cells supplemented with ferrous sulfate (50 µM) or PBS. **G** TET enzymatic activity in R1-WT cells following siRNA-mediated knockdown of *Fthl17c*. **H** Dot blot and 5mc ELISA analysis of 5mC levels in *Fthl17c* knockdown R1-WT cells versus siRNA control. **I** TET enzymatic activity in R1-C5 cells after *Fthl17c* overexpression versus vector control. **J** Dot blot and 5mc ELISA analysis of 5mC levels in R1-C5 cells overexpressing *Fthl17c* or empty vector. All quantification data represent mean ± SD from three independent experiments. Unpaired two-tailed t tests (**B**, **D**, **G**-**J**) and one-way ANOVA (**E**, **F**) were used to determine statistical significance. *p < 0.05, **p < 0.01, ***p < 0.001, ****p < 0.0001; ns, not significant

Ferrous iron (Fe^2^⁺) serves as an essential cofactor for Fe^2^⁺/α-ketoglutarate dependent dioxygenases, including ten eleven translocation (TET) enzymes that catalyze the conversion of 5-methylcytosine (5mC) to 5-hydroxymethylcytosine (5hmC) (Fig. 4A). Given the elevated global DNA methylation levels observed in R1-C5 cells compared with R1-WT cells (Chen et al. 2023), we next asked whether impaired TET enzymatic activity contributes to this phenotype.

We first directly assessed TET enzymatic activity. Quantitative TET activity assays revealed a significant reduction in total TET activity in R1-C5 cells relative to R1-WT controls (Fig. 4B), indicating compromised DNA demethylation capacity upon loss of MSI1/MSI1-C. To determine whether this reduction reflected altered enzyme abundance, we next examined TET protein expression levels. Western blot analysis showed comparable protein levels of TET1 and TET2 in R1-WT and R1-C5 cells (Fig. 4C), suggesting that diminished catalytic activity, rather than reduced protein expression, underlies the observed hypermethylation phenotype.

To assess whether reduced iron availability was sufficient to impair TET activity, R1-WT cells were treated with 12.5 µM iron chelator deferoxamine (DFO) during serial passaging. Compared with DMSO treated controls, DFO treated cells showed progressively reduced expression of pluripotency markers *Pou5f1* and *Nanog* (Fig. S4A). Consistently, DFO treated colonies gradually lost the compact dome shaped morphology characteristic of undifferentiated ESCs and displayed differentiated morphology after 5 passages (Fig. S4B).

Consistent with these changes in pluripotency status, dot blot and 5mc ELISA analysis demonstrated that DFO treatment markedly increased global 5mC levels (Fig. 4D). Mechanistically, TET enzymatic activity assays revealed that DFO treatment significantly reduced TET activity (Fig. S4C).

In parallel, treatment of R1-C5 cells with 100 µM vitamin C (L-ascorbic acid) resulted in a significant reduction in 5mC abundance (Fig. 4E) and increased expression of pluripotency associated genes (Fig. S4D) and elevated TET enzymatic activity (Fig. S4E). In contrast, direct addition of ferrous sulfate failed to reduce 5mC abundance (Fig. 4F) or restore TET enzymatic activity (Fig. S4F).

We next directly tested the contribution of *Fthl17c* to TET activity. Knockdown of *Fthl17c* in R1-WT cells led to a pronounced decrease in TET enzymatic activity (Fig. 4G) accompanied by an increase in global 5mC levels (Fig. 4H). Conversely, *Fthl17c* overexpression in R1-C5 cells restored TET activity (Fig. 4I) and reduced DNA methylation levels (Fig. 4J).

To determine whether this effect depends on iron availability, *Fthl17c* overexpressing R1-C5 cells were treated with DFO. DFO treatment markedly reduced TET enzymatic activity and increased global 5mC levels, reversing the effects of *Fthl17c* overexpression (Fig. S4G). These results indicate that *Fthl17c* regulates TET activity in an iron dependent manner.

Together, these results establish a direct association between *Fthl17c* expression, intracellular Fe^2^⁺ availability, TET enzymatic activity, and global DNA methylation levels in embryonic stem cells.

### FTHL17C associates with TET1 in the nucleus of embryonic stem cells

**Fig. 5:**
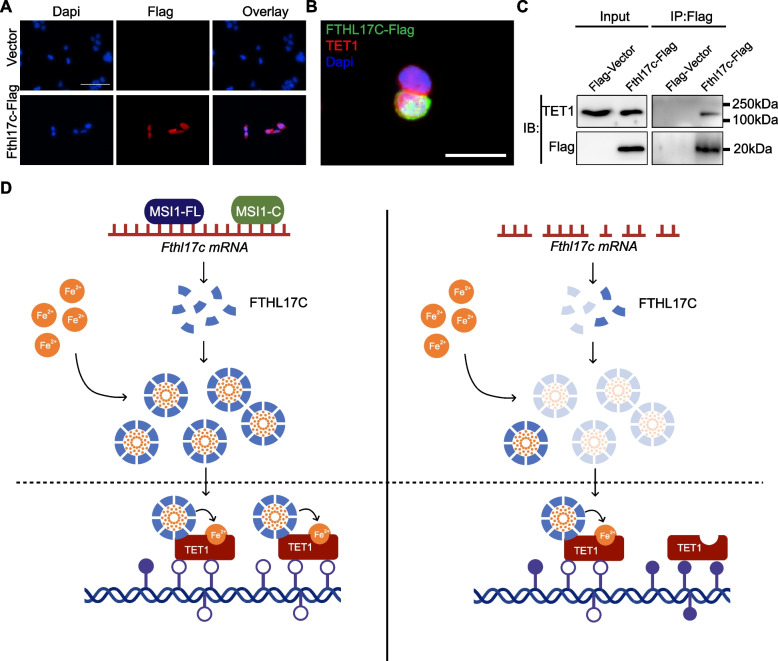
FTHL17C interacts with TET1 to support nuclear Fe^2^⁺ delivery and DNA demethylation. **A** Immunofluorescence analysis showing that FTHL17C-Flag localizes to both the cytoplasm and nucleus in overexpressing R1-WT cells. **B** Co-immunofluorescence staining reveals partial nuclear co-localization between Fthl17C-Flag and endogenous TET1 in R1 cells. **C** Co-immunoprecipitation (co-IP) using anti-Flag magnetic beads confirms a physical interaction between FTHL17C and TET1 in R1 cells. Flag-Vector as negative control. **D** Schematic model illustrating the proposed MSI1-FTHL17C-Fe^2^⁺-TET regulatory axis that maintains DNA hypomethylation and ESC pluripotency

To investigate how FTHL17C is positioned to regulate TET enzymatic activity, we first examined its subcellular localization. Immunofluorescence analysis of ESCs overexpressing FTHL17C-Flag revealed signals in both the cytoplasm and nucleus (Fig. 5A). Immunofluorescence co-staining showed that FTHL17C-Flag partially co-localized with TET1 within the nucleus of R1 cells (Fig. 5B), suggesting a potential interaction between the two proteins.

To test this interaction biochemically, *Fthl17c*-Flag was overexpressed in R1-WT cells, and protein complexes were captured using anti-Flag magnetic beads. Western blot analysis of the eluted proteins revealed the presence of endogenous TET1 among the FTHL17C associated partners (Fig. 5C), confirming that FTHL17C physically interacts with TET1. Given that FTHL17C belongs to a ferritin like family capable of binding Fe^2^⁺, this interaction suggests that FTHL17C may serve as a carrier or donor facilitating the delivery of ferrous ions to nuclear TET enzymes. Interestingly, although immunofluorescence analysis also revealed partial co-localization between FTHL17C-Flag and TET2 (Fig. S5A), subsequent co-immunoprecipitation failed to detect interaction between FTHL17C and TET2 (Fig. S5B). This suggests that the association with TET2, if present, may be transient, weak, or indirect under the tested conditions.

Collectively, these findings support a mechanistic model in which MSI1-FL and MSI1-C bind to and stabilize *Fthl17c* mRNA, leading to sustained FTHL17C protein expression. FTHL17C, in turn, binds Fe^2^⁺ and interacts with TET1 in the nucleus, likely facilitating Fe^2^⁺ delivery required for TET catalytic activity and maintaining global DNA demethylation required for ESC pluripotency. In contrast, loss of MSI1-FL/MSI1-C destabilizes *Fthl17c* mRNA and reduces FTHL17C protein levels, resulting in impaired Fe^2^⁺ delivery to TET1, diminished TET catalytic activity, increased DNA methylation, and consequent loss of pluripotency (Fig. 5D).

## Discussion

Our study identifies an MSI1-FTHL17C-Fe^2^⁺-TET-5hmC regulatory axis that supports embryonic stem cell (ESC) pluripotency. We show that the RNA binding protein Musashi-1 (MSI1), particularly its short C-terminal isoform MSI1-C, maintains the pluripotent state by post transcriptionally stabilizing *Fthl17c* mRNA. Sustained *Fthl17c* expression preserves intracellular ferrous iron availability, which is required for efficient TET dioxygenase activity and the generation of 5-hydroxymethylcytosine (5hmC). Disruption of MSI1/MSI1-C or FTHL17C impairs Fe^2^⁺ homeostasis, reduces TET activity, increases DNA methylation, and leads to loss of pluripotency, highlighting the importance of this pathway in maintaining the hypomethylated epigenetic landscape characteristic of ESCs.

Mechanistically, our data support a model in which MSI1-C binds and stabilizes *Fthl17c* transcripts, ensuring sustained production of a ferritin like protein that preserves iron in a bioavailable Fe^2^⁺ state. Unlike canonical ferritin heavy chains, FTHL17C lacks ferroxidase activity, consistent with a role distinct from long term iron sequestration. We further show that FTHL17C localizes to the nucleus and associates with TET1, providing a potential route for supplying Fe^2^⁺ to Fe^2^⁺ dependent epigenetic enzymes. This framework explains why global iron supplementation alone is insufficient to restore TET activity in MSI1/MSI1-C deficient cells, whereas vitamin C, by maintaining iron in a reduced state, effectively rescues DNA demethylation.

Beyond defining a specific molecular pathway, our findings reveal a broader layer of pluripotency regulation. While ESC identity has traditionally been framed around transcriptional networks and signaling pathways, our results demonstrate that RNA-binding proteins can influence cell fate by controlling the availability of metabolic cofactors required for epigenetic modification. This post-transcriptional regulation of iron metabolism provides a direct link between cellular metabolic state and chromatin regulation. Given that many chromatin modifying enzymes belong to the Fe^2^⁺ dependent dioxygenase family, similar regulatory principles may extend beyond TET mediated DNA demethylation.

Our work also raises questions regarding species-specific implementation of this pathway. In mice, *Fthl17* comprises a clustered multigene family, with *Fthl17b-f* showing high sequence conservation, whereas *Fthl17a* is more divergent and may serve a distinct function. In contrast, humans possess a single *FTHL17* gene. Notably, MSI1-C is expressed in naïve-state human ESCs but is largely absent in primed pluripotent states, suggesting that the MSI1-FTHL17 axis may represent a conserved module associated with primordial pluripotency. Whether human FTHL17 similarly regulates nuclear Fe^2^⁺ availability and TET activity remains an important question for future studies.

In conclusion, our findings complete a previously missing dimension of MSI1 function in stem cell biology. Although MSI1 was originally named after Miyamoto Musashi, the legendary swordsman renowned for wielding two blades, functional studies of MSI1 have historically focused on a single sword the full length, RRM containing protein. Our work, together with our earlier identification of MSI1-C, demonstrates that the MSI1 locus indeed encodes a dual regulatory system. Here, we reveal that the short C-terminal isoform MSI1-C constitutes the second functional blade, operating through post-transcriptional regulation of iron metabolism to sustain Fe^2^⁺-dependent TET activity and DNA demethylation. By uncovering how MSI1-C links RNA regulation, metabolic cofactor availability, and epigenetic remodeling, this study not only completes the functional narrative of MSI1 but also establishes a broader principle: stem cell identity is safeguarded by coordinated control of gene expression and metabolic epigenetic coupling. This dual layered regulatory architecture may represent a general strategy by which stem and progenitor cells preserve developmental potential across embryonic and regenerative contexts.

## Method

### Cell culture

R1 mouse embryonic stem cells (mESCs) were purchased from ATCC. R1 mESCs were cultured in dishes coated with 0.2% gelatin or in dishes containing feeder cells. Mouse embryonic stem cell medium (knockout DMEM supplemented with 15% knockout serum analog, 1 × GlutaMAX, 1 × Antibiotic–Antimycotic, 1 × MEM Non-Essential Amino Acids, 0.1 mM 2-Mercaptoethanol, and 1000 Units/mL ESGRO-mLIF) was used to culture R1 mESCs at 37 °C under 5% CO₂ conditions. R1 mouse embryonic stem cells were passaged as single cells using 0.05% trypsin–EDTA (Life Technologies). The generation and validation of the MSI1/MSI1-C knockout R1-C5 cell line using CRISPR/Cas9, including sgRNA design and knockout verification, were previously described (Chen et al. 2023). During R1-C5 cell passaging, cells were dissociated into single cells and placed in gelatin coated culture dishes and cultured at 37 °C, 5% CO₂ for 45 min to allow differentiated cells to adhere (eliminating non target cells during R1-C5 passaging), followed by collection of suspension cells for passaging. All cell lines tested negative for mycoplasma and underwent identification and validation. To model iron limitation, R1 cells were treated with 12.5 µM deferoxamine (DFO) during long term passaging, and the concentrations of DFO were determined by titration experiments. In rescue experiments, R1-C5 cells were treated with 100 µM L-ascorbic acid (vitamin C), which was freshly prepared before used.

### siRNA transfection

siRNA sequences are listed in Supplementary Table S2. For siRNA transfection, Lipofectamine 3000 (Thermo Fisher) Reagent was used. Briefly, R1 mESCs were passaged as single cells using 0.05% trypsin–EDTA (Life Technologies). Then, 1 × 10^6^ cells were seeded into one well of a 6-well plate pre-coated with gelatin and cultured in mouse ESC medium. The following day, the medium was replaced, and 30 pmol of siRNA mixed with 3 µL of Lipofectamine 3000 was added to the cells. Cells were used for subsequent analysis 48 h after siRNA transfection.

### Western blotting

Cells were collected and homogenized with RIPA buffer (Thermo Scientific) containing complete protease inhibitors for protein extraction. Cell lysate was denatured by adding 6 × protein loading buffer (Beyotime) and heated for 10 min at 90 °C. Proteins were separated on SDS-polyacrylamide gels and processed for conventional western blotting. The primary and secondary antibodies were diluted in TBST (Tris: 20 mM, NaCl: 150 mM, Tween 20 detergent: 0.1% w/v) containing 5% (w/v) skimmed milk. Peroxidase activity was detected by chemiluminescence (Vazyme, SuperFemto ECL Chemiluminescence Kit) and captured with Amersham Imager 600 (General Electric). The antibodies used are listed in the key resources table (Table S1).

### Immunofluorescence staining

Cells were fixed in 4% paraformaldehyde (Sigma-Aldrich) and permeabilized with 1% Triton X-100 (Sigma-Aldrich) in PBS for 10 min. After blocking with blocking buffer (PBS containing 5% BSA and 0.3% Triton X-100), cells were incubated with the primary antibody, washed three times with PBS, and incubated with secondary antibodies (1:500–1000 dilution) followed by counterstaining with DAPI. Immunofluorescence data were captured using DMI-6000B microscopes equipped with digital cameras (Leica). The antibodies used are listed in the key resources table (Table S1).

### Dot blot assay for 5mC

The mESCs were collected using the differential adhesion method to remove feeder and differentiated cells, and then extracted using the Genome Extraction Kit (Tiangen). DNA concentration was determined using Nanodrop (Thermo Fisher), and DNA was diluted to 250 ng/µL, followed by gradient dilution to 125 ng/µL and 62.5 ng/µL. 2 µL of each concentration were spotted on a nylon membrane, followed by UV cross-linking for 15 min. The membrane was then incubated in 5% skim milk for 2 h for blocking. Anti-5mC antibodies and secondary antibodies (Table S1) were diluted in TBST (Tris: 20 mM, NaCl: 150 mM, Tween 20 detergent: 0.1% w/v) containing 5% (w/v) skimmed milk. Peroxidase activity was detected by chemiluminescence using ECL Western blot substrate (Vazyme) and captured with Amersham Imager 600 (General Electric).

### Global DNA methylation ELISA

Global DNA methylation was measured using the MethylFlash™ Global DNA Methylation (5-mC) ELISA Easy Kit (Colorimetric) (EpigenTek) according to the manufacturer’s instructions. Genomic DNA was extracted from the indicated samples, and 100 ng DNA per well was used for the assay. Samples were sequentially incubated with the 5-mC capture antibody and detection antibody, followed by colorimetric signal development. Absorbance was measured at 450 nm using a microplate reader. Global DNA methylation levels were calculated according to the manufacturer’s protocol.

### RNA stability assay

R1 mESC were cultured in 0.2% gelatin-coated dishes for three generations to remove Feeder cells thoroughly. 6 × 10^5^ cells were seeded into 6-well plates. Cells were collected after 24 h utilizing a cell scraper, and centrifuged at 500 g for 3 min to remove the supernatant. Cells were lysed by the addition of Freezol and frozen at −80 °C as an initial sample (t = 0), followed by the addition of Actinomycin D at a final concentration of 20 µg/mL in the remaining 5 wells, and the cells were collected at time points 1, 2, 4, 6, and 8 h after addition of Actinomycin D and the cell lysed in Trizol as described above. RNA was extracted according to the instructions of Freezol reagent. After removing genomic DNA using DNAase, 1 µg of RNA was used for reverse transcription of cDNA for qPCR analysis.

### RT-qPCR

Total RNA was extracted using FreeZol Reagent (Vazyme), and reverse-transcribed into cDNA using HiScript III RT SuperMix for qPCR kit (Vazyme). ChamQ Universal SYBR qPCR Master Mix (Vazyme) was used for RT-qPCR and detection using Lightcycler (Roche). Primers are listed in Table S2.

### DNA constructs

Fragments of *Fthl17c* genes were obtained by PCR amplification of cDNA reverse-transcribed from total RNA obtained from R1 mESCs, using primers listed in Table S2. PCR products were purified and TA cloned into *pEASY-T1* (TransGen). After sequencing, the *Fthl17c-Flag* was digested with *BamH*I and *EcoR*V and subcloned into pCDNA3.1 (*BamH*I/*EcoR*V, Thermo Fisher Scientific) and the lentivirus vector CSll-EF-MSC-2A-Neo (*BamH*I/*Hpa*I). The expression of DNA was confirmed by Western blot analysis of Flag.

### In vitro transcription of RNA and 3' end biotin labeling

Using the PCDNA3-Fthl17c plasmid as a template, amplify the T7-Fthl17c DNA fragment (approximately 550 bp) via PCR. Primer sequences are provided in Table S2. After purifying the DNA fragment, transcribe *Fthl17c* RNA in vitro using an in vitro transcription kit with 0.5 µg of the DNA fragment. Subsequently, RNA purification was performed using the MEGAclear™ Transcription Clean-Up Kit. Ten micrograms of RNA (approximately 50 pmol) were biotinylated at the 3' end using the RNA 3' End Biotinylation Kit according to the manufacturer's instructions. The purified biotinylated at the 3' end *Fthl17c* RNA was used for subsequent experiments.

### RNA pull-down assay

Cultured 293FT cells in 10 cm cell culture dishes. Upon reaching 60% confluence, cells were transfected with PCDNA3-MSI1-FL-Flag or PCDNA3-MSI1-C-Flag plasmids using Lipofectamine 3000. After 48 h of overexpression, transfect 10 µg of 3'-terminally biotinylated Fthl17c RNA into 293FT cells overexpressing MSI1-FL-Flag or MSI1-C-Flag using Lipofectamine 3000 reagent. Four hours post-transfection, aspirate the medium, wash cells three times with pre-chilled PBS, and place on ice for UV crosslinking at 254 nm with 0.15 J/cm^2^. After crosslinking, add 5 mL ice-cold 90% ethanol to fix cells for 30 min. Cells were then lysed in lysis buffer (20 mM Tris–HCl, pH 7.5, 500 mM LiCl, 1 mM EDTA pH 8.0, 0.5% lithium dodecyl sulfate (LiDS), and 5 mM DTT containing a protease inhibitor cocktail and ribonuclease inhibitor) and collected by scraping. Homogenize the lysate using a syringe equipped with a 0.4 mm needle. Use 5% of the lysate as the input control. RNA-protein complexes were isolated using streptavidin magnetic beads by continuous rotation incubation with the lysate for 2 h. Beads were separated on a magnetic stand and washed with lysis buffer and Buffer 1 (20 mM Tris–HCl, pH 7.5, 500 mM LiCl, 1 mM EDTA pH 8.0, 0.1% LiDS, 5 mM DTT). Buffer 1 (20 mM Tris–HCl pH 7.5, 500 mM LiCl, 1 mM EDTA pH 8.0, 0.1% LiDS, 5 mM DTT), Buffer 2 (20 mM Tris–HCl pH 7.5, 500 mM LiCl, 1 mM EDTA pH 8.0, and 5 mM DTT), and Buffer 3 (20 mM Tris–HCl pH 7.5, 200 mM LiCl, 1 mM EDTA pH 8.0, and 5 mM DTT). Each buffer was applied for two washes under rotation conditions (10 min at 4 °C). To extract proteins, resuspend magnetic beads in 100 µL of protein elution buffer (20 mM Tris pH 7.0, 1 mM DTT, and 1% SDS). Incubate with 100 U RNase A at 37 °C for 1 h. Subsequently, boil the sample for 5 min and place it on ice for subsequent Western blot analysis.

### Weighted gene co-expression network analysis (WGCNA)

Weighted gene co-expression network analysis (WGCNA) was performed on normalized transcriptomic data from R1-WT and R1-C5 cells to identify gene modules associated with loss of pluripotency in MSI1-deficient ESCs. Pairwise gene correlations were used to construct an unsigned co-expression network with a soft-thresholding power of 28. Modules were identified by hierarchical clustering of the topological overlap matrix, followed by dynamic tree cutting (minimum module size = 30 genes). Module eigengenes were correlated with sample traits to identify co-expression modules associated with the differentiated state.

### Measurement of intracellular Ferrous Iron (Fe^2^⁺) by flow cytometry

To quantify intracellular labile ferrous iron (Fe^2^⁺), cells were harvested and incubated with 5 µM RhoNox fluorescent probe (Beyotime) in complete medium at 37 °C for 30 min, protected from light. Following incubation, cells were washed twice with PBS and immediately analyzed using a flow cytometer (BD). Fluorescence was detected in the PE channel (excitation: 561 nm; emission: 585/15 nm). Forward scatter (FSC) and side scatter (SSC) gates were applied to exclude debris, and single cells were selected using FSC-H versus FSC-A plots. At least 10,000 live cells were acquired per sample. Data were analyzed using FlowJo software, and mean fluorescence intensity (MFI) of RhoNox was quantified for each condition.

### Measurement of nuclear Ferrous Iron (Fe^2+^) by RhoNox

ESC colonies were dissociated into single cells using Accutase. Cells were incubated with 5 µM RhoNox fluorescent probe together with Hoechst nuclear stain to label nuclei. Cell suspensions were mounted onto microscope slides and imaged using a fluorescence microscope. Fluorescence intensities of RhoNox and Hoechst were quantified using ImageJ software, and nuclear Fe^2^⁺ levels were determined by calculating the RhoNox/Hoechst fluorescence intensity ratio for each cell.

### Nuclear and cytoplasmic protein extraction

Nuclear and cytoplasmic proteins were isolated using a Nuclear Protein Extraction Kit (Beyotime) according to the manufacturer’s instructions. Cells were first lysed on ice and centrifuged to collect the pellet. The pellet was subsequently resuspended in nuclear extraction reagent to obtain the nuclear protein fraction. Protein concentrations were determined and total iron was measured using Iron Assay Kit (Sigma-Aldrich, MAK472).

### Measurement of total intracellular iron content

Total intracellular iron was quantified using the Iron Assay Kit (Sigma-Aldrich, MAK472) according to the manufacturer’s instructions. Briefly, cells were harvested and lysed, and 50 µL of each sample was transferred to a clear 96-well plate. Iron standards ranging from 0 to 1000 µg/dL were prepared in parallel. A freshly prepared working reagent (200 µL per well, containing reagents A, B, and C) was added to each sample and standard well. After incubation at room temperature for 40 min, absorbance was measured at 590 nm using a microplate reader. Total iron concentration was calculated from the standard curve and normalized to protein content.

### TET enzyme activity assay

TET enzymatic activity was measured using the Epigenase™ 5mC Hydroxylase TET Activity/Inhibition Assay Kit (Epigentek) according to the manufacturer’s instructions. Briefly, 5-10 µg of nuclear extract from mouse ESCs was added to assay wells pre-coated with methylated DNA substrate. The enzymatic reaction was initiated by adding reaction buffer containing cofactors and incubating the plate at 37 °C for 90 min. Active TET enzymes catalyzed the conversion of 5mC to 5hmC, which was then detected using a capture antibody specific to 5hmC, followed by a detection antibody and enhancer solution. After final colorimetric development, absorbance was measured at 450 nm using a microplate reader. All samples were assayed in technical duplicates. TET activity was calculated as OD per minute per µg of input protein, normalized to protein concentration as measured by the BCA assay.

### Statistical analyses

All statistical analyses were performed using GraphPad Prism 9 (GraphPad Software). Data are presented as mean ± standard deviation (SD) from at least three independent experiments. Statistical significance between two groups was determined using unpaired two-tailed Student’s t-tests. For comparisons involving more than two groups, one-way analysis of variance (ANOVA) was applied, as indicated in the figure legends. Asterisks denote levels of statistical significance: * indicates *P* < 0.05, ** indicates *P* < 0.01, *** indicates *P* < 0.001, **** indicates *P* < 0.0001.

## Supplementary Information

Supplementary Material 1. Figure S1. Transcriptomic evidence of pluripotency loss and endodermal bias in MSI1/MSI1-C-deficient ESCs. Figure S2. Sequence similarity and expression profiling of Fthl17 gene family in mouse ESCs. Figure S3. Fthl17c regulates nuclear ferrous iron availability. Figure S4. Vitamin C restores pluripotency marker expression in MSI1/MSI1-C-deficient ESCs. Figure S5. Subcellular localization and interaction specificity of FTHL17C with TET proteins. Table S1. Key resources table. Table S2. Oligonucleotide used in the current study.

## Data Availability

RNA sequencing (RNA-seq) and RNA immunoprecipitation sequencing (RIP-seq) data analyzed in this study were previously generated and published (Chen et al. 2023) and available in the Gene Expression Omnibus (GEO) database under accession number GSE197608.

## Abbreviations

ESC: Embryonic stem cell
mESC: Mouse embryonic stem cell
MSI1: Musashi-1
MSI1-C: Musashi-1 C-terminal isoform
FTHL17C: Ferritin-like heavy chain protein 17C
TET: Ten-eleven translocation dioxygenase
5mC: 5-Methylcytosine
5hmC: 5-Hydroxymethylcytosine
Fe^2^⁺: Ferrous iron
RIP-seq: RNA immunoprecipitation sequencing
WGCNA: Weighted gene co-expression network analysis
GSEA: Gene set enrichment analysis
DFO: Deferoxamine
