# Supplementary material for: MSI1-FTHL17C-iron circuit couples metabolic and epigenetic control of pluripotency in mouse embryonic stem cells
Source: Cell Regen. 2026 May 6;15:14. doi: 10.1186/s13619-026-00288-8 (PMC13149808; doi:10.1186/s13619-026-00288-8)
Supplement: Supplementary file 1 — Supplementary Material 1. Figure S1. Transcriptomic evidence of pluripotency loss and endodermal bias in MSI1/MSI1-C-deficient ESCs. Figure S2. Sequence similarity and expression profiling of Fthl17 gene family in mouse ESCs. Figure S3. Fthl17c regulates nuclear ferrous iron availability. Figure S4. Vitamin C restores pluripotency marker expression in MSI1/MSI1-C-deficient ESCs. Figure S5. Subcellular localization and interaction specificity of FTHL17C with TET proteins. Table S1. Key resources table. Table S2. Oligonucleotide used in the current study. [file 13619_2026_288_MOESM1_ESM.docx]

**Supplemental Information**

**
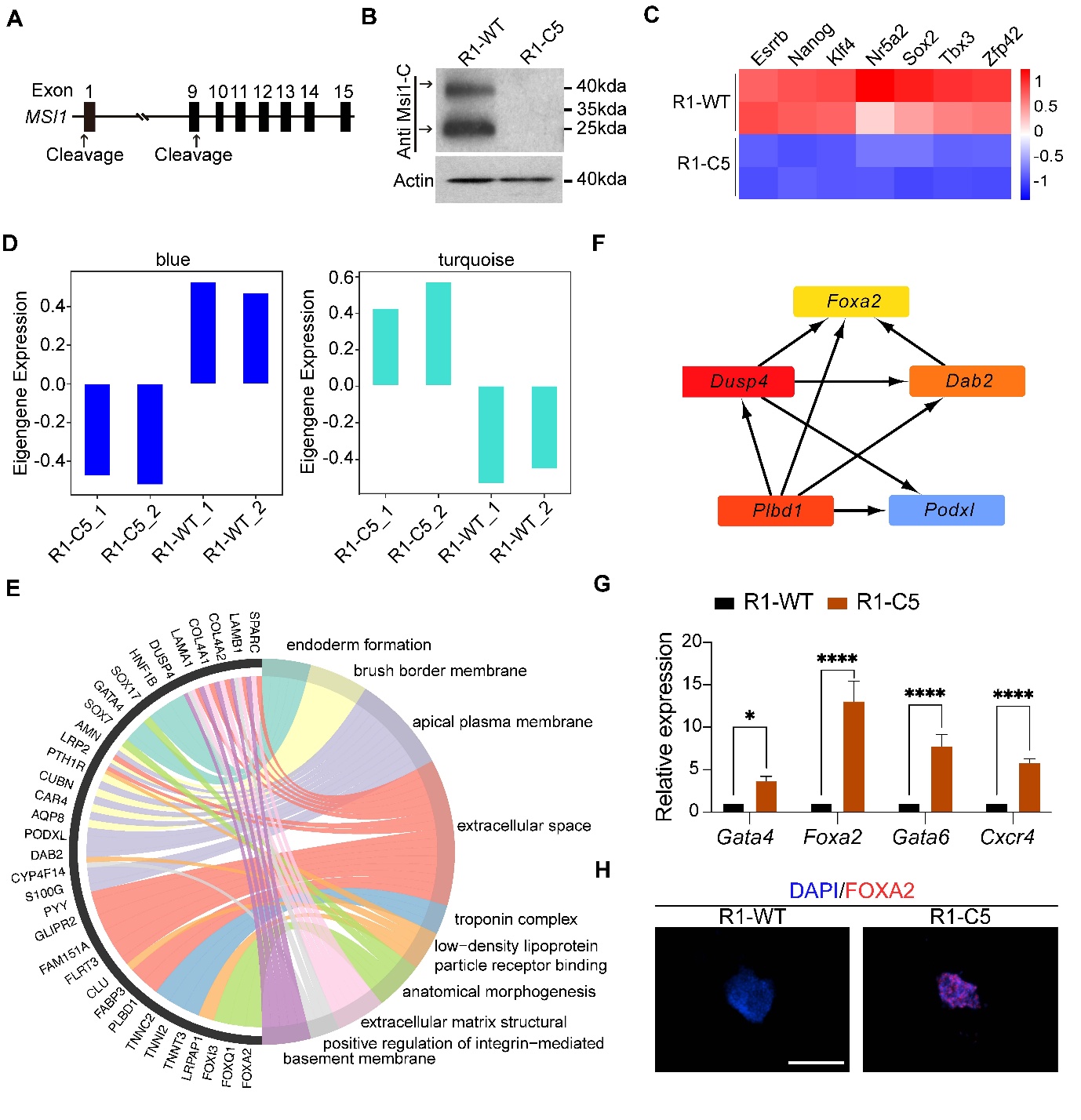
**

**Figure S1. Transcriptomic evidence of pluripotency loss and endodermal bias in MSI1/MSI1-C-deficient ESCs. Related to Figure 1.**

(A) Schematic representation of Msi1 gene knockout strategy used to generate the R1-C5 cell line.

(B) Western blot analysis of MSI1-FL and MSI1-C protein expression in R1-WT and R1-C5 cells.

(C) Heatmap showing relative expression levels of pluripotency-associated genes (*Esrrb*, *Nanog*, *Klf4*, *Nr5a2*, *Sox2*, *Tbx3*, *Zfp42*) in R1-WT versus R1-C5 cells.

(D) Module eigengene expression of blue and turquoise modules from WGCNA analysis, showing opposing trends between R1-WT and R1-C5 cells.

(E) GO enrichment analysis of genes in the turquoise module, highlighting pathways related to endodermal differentiation.

(F) Network analysis identifying hub genes (e.g., *Dusp4*, *Plbd1*, *Foxa2*) in the turquoise module.

(G) RT-qPCR quantification of endoderm marker genes (*Gata4*, *Foxa2*, *Gata6*, *Cxcr4*) in R1-WT and R1-C5 cells. Data are shown as mean ± SD; unpaired two-tailed t test; *p < 0.05, ****p < 0.0001.

(H) Immunofluorescence staining of FOXA2 in R1-WT and R1-C5 cells. Scale bars, 100 μm.


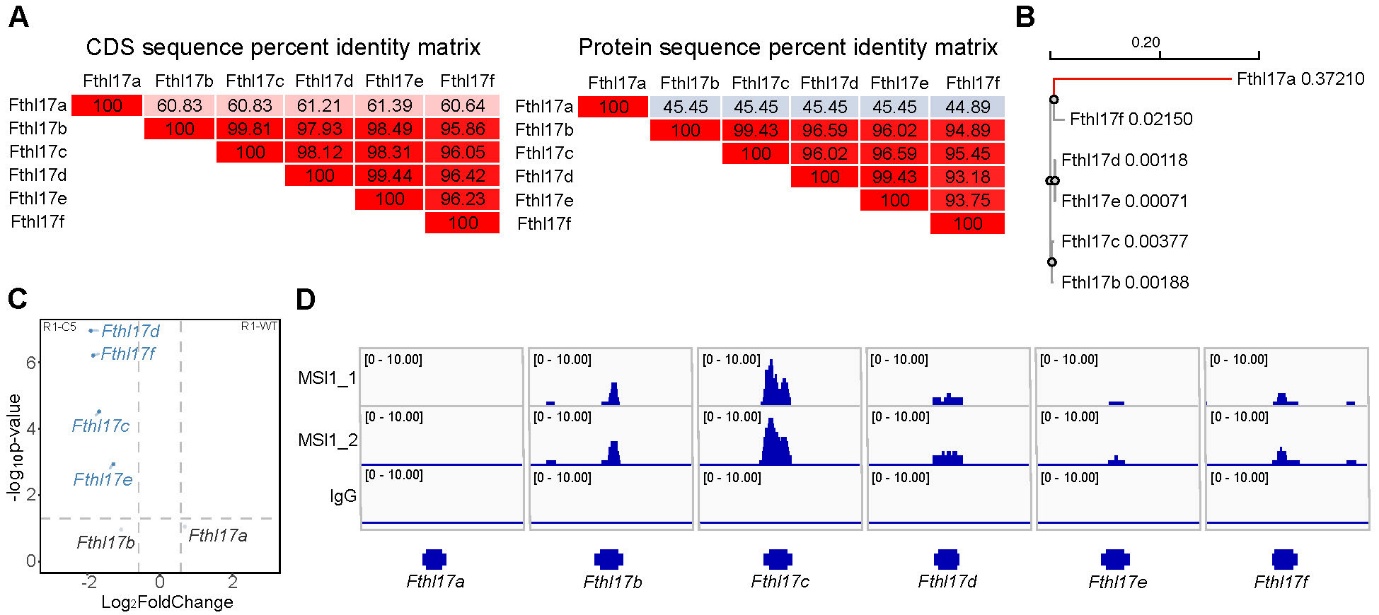


**Figure S2. Sequence similarity and expression profiling of Fthl17 gene family in mouse ESCs. Relative to Figure2.**

(A) Percent identity matrix of coding DNA and protein sequences among the six mouse *Fthl17* paralogs.

(B) Phylogenetic tree of *Fthl17* family members based on protein sequence alignment, showing that *Fthl17a* is more divergent than the other paralogs.

(C) Volcano plot of RNA-seq data comparing expression levels of Fthl17 family genes between R1-WT and R1-C5 cells. *Fthl17c-f* are significantly downregulated in R1-C5.

(D) Integrated Genome Viewer (IGV) tracks GV of previously published MSI1 RIP-seq data (Chen et al., 2023) MSI1 RIP-seq binding peaks across *Fthl17* family loci, with the strongest peak at *Fthl17c*.


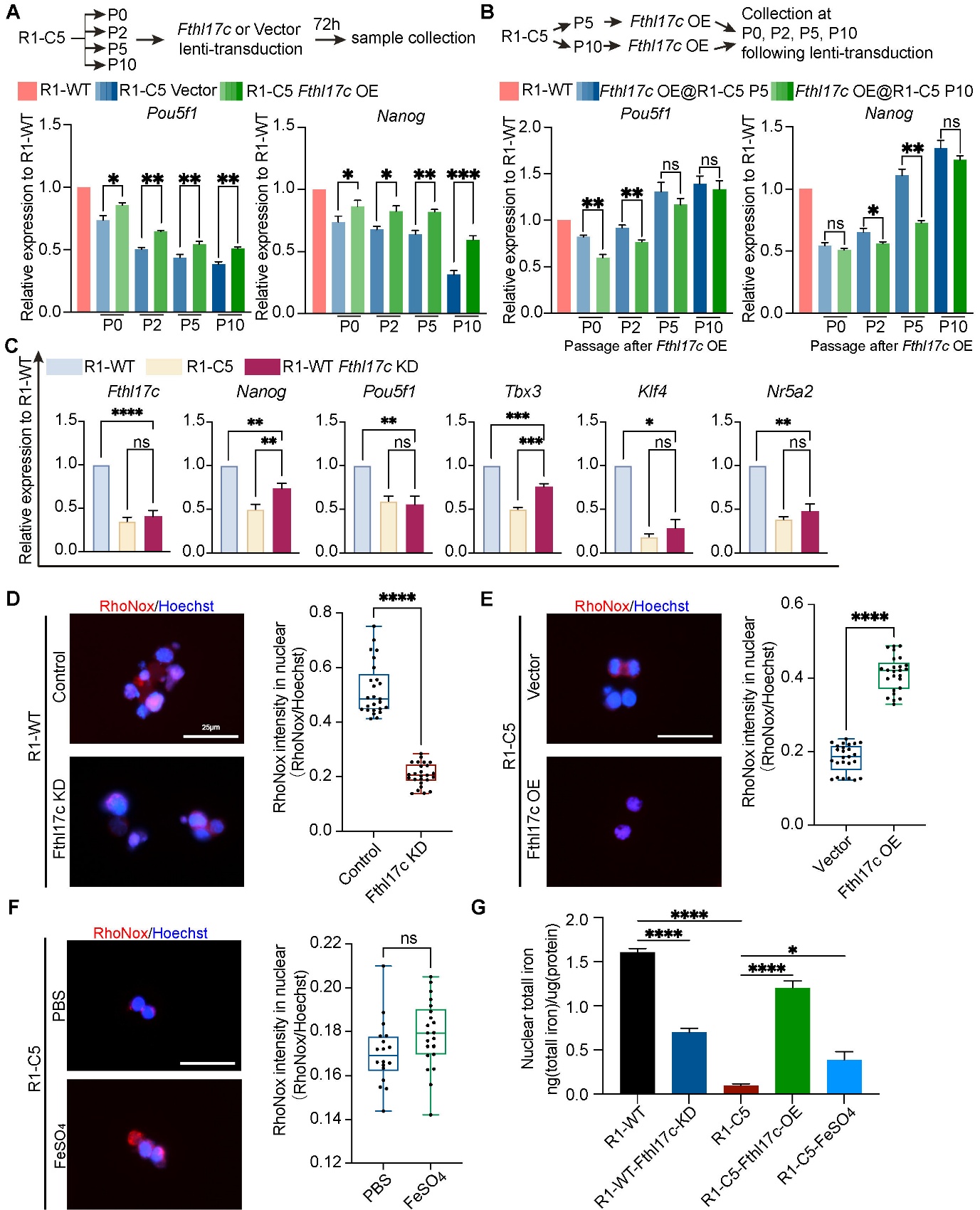


**Figure S3. Fthl17c regulates nuclear ferrous iron availability. Relative to Figure 3.**

(A) RT-qPCR analysis of *Pou5f1* and *Nanog* expression in R1-WT cells and in R1-C5 cells transduced with Fthl17c or vector at different passages (P0, P2, P5, and P10). Samples were collected 72 h after lentiviral transduction.

(B) RT-qPCR analysis of *Pou5f1* and *Nanog* expression in R1-WT cells and in R1-C5 cells in which Fthl17c overexpression was initiated at P5 or P10, followed by serial passaging and sample collection at P0, P2, P5, and P10 after lentiviral transduction.

(C) RT-qPCR analysis of *Fthl17c* and pluripotency marker genes (*Nanog,* *Pou5f1*, *Tbx3*, *Klf4*, *Nr5a2*) in R1-WT, R1-C5, R1-WT with *Fthl17c* knockdown.

(D-F) Representative fluorescence images of RhoNox-1 (red, ferrous iron) and Hoechst (blue, nuclei) in R1-WT cells with *Fthl17c* knockdown (D), R1-C5 cells with *Fthl17c* overexpression (E), and R1-C5 cells treated with FeSO_4_ (F) Quantification of nuclear RhoNox intensity normalized to Hoechst. Scale bar, 25 µm.

(G) Quantification of total intracellular iron in R1-WT, R1-C5, *Fthl17c* overexpressing R1-C5 cells and R1-C5 cells treated with FeSO_4_.

All data represent mean ± SD from three independent experiments. Statistical significance was assessed using unpaired two-tailed t tests (A-C and D-G) and one-way ANOVA (C). *p < 0.05, **p < 0.01, ***p < 0.001, ****p < 0.0001; ns, not significant.


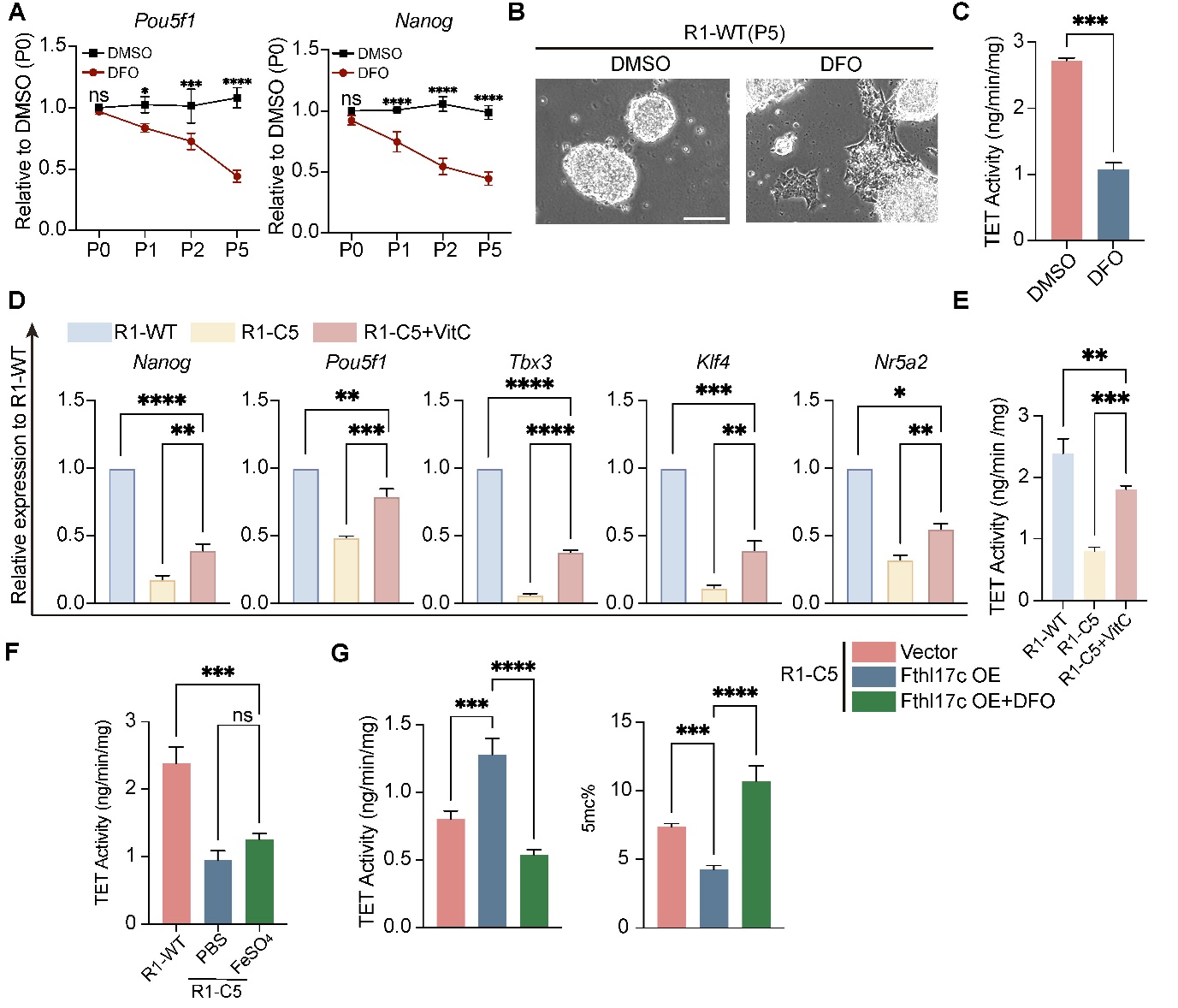


**Figure S4. Vitamin C restores pluripotency marker expression in MSI1/MSI1-C-deficient ESCs. Related to Figure 4.**

(A) RT-qPCR analysis of pluripotency gene expression (*Pou5f1* and *Nanog)* in R1-WT cells treated with DMSO or DFO during serial passaging, analyzed at P0, P2, P5, and P10.

(B) Representative phase-contrast images of R1-WT cells at passage 5 (P5) following serial passaging with DMSO or DFO treatment. Scale bar, 50 μm.

(C) Measurement of TET enzymatic activity in R1-WT cells at passage 5 (P5) after serial passaging with DMSO or DFO treatment.

(D) RT-qPCR analysis of pluripotency genes (*Nanog*, *Pou5f1*, *Tbx3*, *Klf4*, and *Nr5a2*) in R1-WT, R1-C5, and R1-C5 cells treated with vitamin C (L-ascorbic acid, 100 μM). Gene expression levels are normalized to R1-WT and presented as mean ± SD (n = 3).

(E) Measurement of TET enzymatic activity in R1-WT, R1-C5 and R1-C5 treatment with 100 μM Vitamin C.

(F) Measurement of TET enzymatic activity in R1-WT, R1-C5, and R1-C5 cells treated with 50 μM FeSO₄.

(G) Measurement of TET enzymatic activity and global 5mC levels (ELISA) in R1-WT, R1-C5, Fthl17c overexpressing R1-C5 cells, and Fthl17c overexpressing R1-C5 cells treated with 12.5 μM DFO.

All data represent mean ± SD from three biological replicates. Statistical significance was assessed using unpaired two-tailed t tests(A, C) and one-way ANOVA(D, F, G). *p < 0.05, **p < 0.01, ***p < 0.001, ****p < 0.0001; ns, not significant


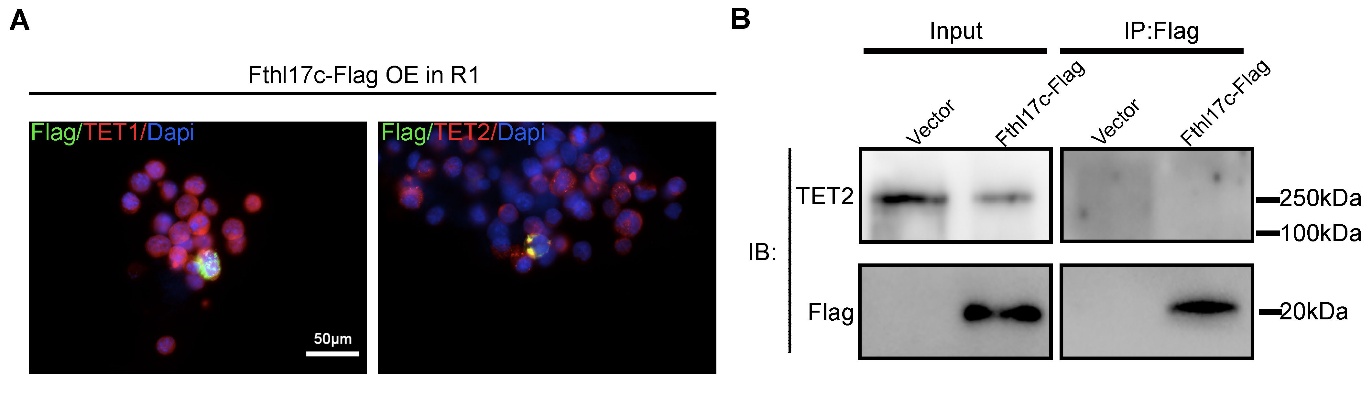


**Figure S5. Subcellular localization and interaction specificity of FTHL17C with TET proteins. Relative to Figure 5.**

(A) Immunofluorescence staining of R1 mESCs overexpressing *Fthl17c*-Flag shows co-localization of Flag (green) with TET1 and TET2 (red) in the nucleus. Nuclei were counterstained with DAPI (blue). Scale bar, 50 μm.

(B) Co-immunoprecipitation (co-IP) performed using anti-Flag magnetic beads in R1 cells reveals no interaction between FTHL17C and TET2.

**Table S1. Key resources table. Related to Method.**

| REAGENT or RESOURCE | SOURCE | IDENTIFIER |
| --- | --- | --- |
| **Antibodies** | | |
| Anti-5-Methylcytosine(5-mc) | ABclonal | Cat# A20599; RRID: AB_3075522 |
| Anti-FLAG | Beyotime | Cat# AF0036; RRID:  AB_3683542 |
| Anti-TET1 | ABclonal | Cat# A23162; RRID:  AB_3668884 |
| Anti-TET2 | ABclonal | Cat# A5682; RRID:  AB_2766442 |
| Donkey anti-Mouse IgG (H+L) Highly Cross-Adsorbed Secondary Antibody, Alexa Fluor 488 | Thermo Fisher Scientific | Cat# A21202 RRID: AB_141607 |
| Donkey anti-Rabbit IgG (H+L) Highly Cross-Adsorbed Secondary Antibody, Alexa Fluor Plus 555 | Thermo Fisher Scientific | Cat# A32794 RRID: AB_2762834 |
| Goat anti-Mouse IgG (H+L) Highly Cross-Adsorbed Secondary Antibody, Alexa Fluor 555 | Thermo Fisher Scientific | Cat# A21424 RRID: AB_2275404 |
| Goat anti-Mouse IgG (H+L) Secondary Antibody, HRP | Thermo Fisher Scientific | Cat# 31430 RRID: AB_228307 |
| Goat anti-Rabbit IgG (H+L) Secondary Antibody, HRP | Thermo Fisher Scientific | Cat# 65-6120 RRID: AB_2533967 |
| **Bacterial and virus strains** | | |
| E.coli Stbl3 Competent Cells | sangon | Cat# B528420 |
| DH5α Competent Cells | sangon | Cat# B528413 |
| **Chemicals, peptides, and recombinant proteins** | | |
| Knockout™ DMEM | Thermo Fisher Scientific | Cat# 10829018 |
| Knockout™ KSR | Thermo Fisher Scientific | Cat# 10828028 |
| GlutaMAX | Thermo Fisher Scientific | Cat# 35050061 |
| MEM Non-Essential Amino Acids | Thermo Fisher Scientific | Cat# 11140050 |
| Antibiotic-Antimycotic | Thermo Fisher Scientific | Cat# 15240112 |
| Trypsin-EDTA (0.5%) | Thermo Fisher Scientific | Cat# 15400054 |
| Recombinant Mouse LIF Protein | Millipore | Cat# ESG1107 |
| BME | Sigma-Aldrich | Cat# M3418 |
| FeSO_4_ ·7H_2_O | MERCK | Cat# F8633 |
| Deferoxamine mesylate | Selleck | Cat# S5742 |
| Actinomycin D | Selleck | Cat# S8964 |
| L-Ascorbic acid | MERCK | Cat# A4403 |
| **Critical commercial assays** | | |
| FreeZol Reagent | Vazyme | Cat# R77-01 |
| FastPure Cell/Tissue DNA Isolation Mini Kit | Vazyme | Cat# DC102-01 |
| ChamQ Universal SYBR qPCR Master Mix | Vazyme | Cat# Q711-02 |
| HiScript III RT SuperMix for qPCR | Vazyme | Cat# R323-01 |
| Iron Assay Kit | Sigma-Aldrich | Cat# MAK472 |
| BCA Protein Assay Kit | Beyotime | Cat# P0012S |
| Red Fluorescent Detection Kit for Cellular Ferrous Ions (RhoNox) | Beyotime | Cat# S1070S |
| RIPA Lysis and Extraction Buffer | Thermo Fisher Scientific | Cat# 89900 |
| Lipofectamine® 3000 Reagent | Thermo Fisher Scientific | Cat# L3000001 |
| MEGAscript™ T7 Transcription Kit Plus | Thermo Fisher Scientific | Cat# A57622-25 |
| Pierce™ RNA 3' End Biotinylation Kit | Thermo Fisher Scientific | Cat# 20160 |
| Streptavidin Magnetic Beads | Beyotime | Cat# P2151-1ml |
| Epigenase™ 5mC Hydroxylase TET Activity/Inhibition Assay Kit | EpigenTek | Cat# P-3086 |
| MethylFlash Global DNA Methylation (5-mC) ELISA Easy Kit (Colorimetric) | EpigenTek | Cat# P-1030-96 |
| **Deposited data** | | |
| RNA-seq and RIP-seq data | Chen et al., 2023 | GEO: GSE197608 |
| Experimental models: Cell lines | | |
| R1 mESC | ATCC | SCRC-1011 |
| R1-C5 | Chen et al., 2023 | N/A |
| **Oligonucleotides** | | |
| See Table S2 | This paper | N/A |
| **Recombinant DNA** | | |
| PCDNA3.1 | Invitrogen™ | Cat# V79020 |
| PCDNA3-MSI1-FL-Flag | Chen et al., 2023 | N/A |
| PCDNA3-MSI1-C-Flag | Chen et al., 2023 | N/A |
| PCDNA3-Fthl17c-Flag | This paper | N/A |
| CSll-EF-Fthl17c-Flag-2A-Neo | This paper | N/A |
| **Software and algorithms** | | |
| Adobe Photoshop | Adobe Systems | http://www.adobe.com/products/photoshop.html |
| ImageJ | Fiji | https://ImageJ.nih.gov/ij/ |
| GraphPad Prism 8 | GraphPad Software, Inc | https://www.graphpad.com/scientific-software/prism/ |
| Excel | Microsoft | https://www.microsoft.com/en-gb/ |
| WGCNA | Horvath and Zhang (2005) | https://cran.r-project.org/web/packages/WGCNA/index.html |
| R v3.6.2 | N/A | https://www.R-project.org/ |
| Integrative Genomics Viewer (IGV) | James et al | https://igv.org |

**Table S2. Oligonucleotide used in the current study. Related to Method.**

| **Name** | **Propose** |  | **Sequence 5’-3’** |
| --- | --- | --- | --- |
| Q-mNanog | detection | Forward | AAATCCCTTCCCTCGCCATC |
|  |  | Reverse | CACTGGTTTTTCTGCCACCG |
| Q-mPou5f1 | detection | Forward | GTGGGGCGGTTTTGAGTAAT |
|  |  | Reverse | AAGGTGTCCCTGTAGCCTCA |
| Q-mTbx3 | detection | Forward | CTACGGGGGAGCAATGGATG |
|  |  | Reverse | TGGCTCTGACGATGTGGAAC |
| Q-mKlf4 | detection | Forward | CTGCTGAGTCCAAGAGCGTG |
|  |  | Reverse | GAACGTGGAGAAGGACGGGA |
| Q-mNr5a2 | detection | Forward | TTTTCCCTTGGGCTGTCACTT |
|  |  | Reverse | GCACGTTTTTCCCGGAGTTG |
| Q-mFthl17c | detection | Forward | ACCAAGTCACACAACTGCCA |
|  |  | Reverse | ATGCAGCCTCCACGCTTATT |
| Q-mGata3 | detection | Forward | TCTACGCTCCTTGCTACTCAG |
|  |  | Reverse | CCCAGGAGAGGGGTCGTTTA |
| Q-mGata4 | detection | Forward | GGAAGTGTGCAGCCGATCC |
|  |  | Reverse | ATCACCCACCGGCTAAAGAAG |
| Q-mGata6 | detection | Forward | GTGCCTCGACCACTTGCTAT |
|  |  | Reverse | CTGATGCCCCTACCCCTGAG |
| Q-mFoxa2 | detection | Forward | ATGCACTCGGCTTCCAGTAT |
|  |  | Reverse | TCACGGAAGAGTAGCCCTCG |
| Q-mCxcr4 | detection | Forward | TGCTGACCCTAAACCCCCAA |
|  |  | Reverse | CGGTACTTGTCCGTCATGCT |
| Q-mGapdh | detection | Forward | TGTGAACGGATTTGGCCGTA |
|  |  | Reverse | ACTGTGCCGTTGAATTTGCC |
| T7 | in vitro transcription | Forward | TAATACGACTCACTATAGG |
| SP6 |  | Reverse | ATTTAGGTGACACTATAGA |
| mFthl17c-siRNA | Knockdown | Sense | GGCAGUGUGAGGAUGCUAU |
|  |  | Anti-sense | AUAGCAUCCUCACACUGCC |
| mFthl17c-F | Plasmid Construction | Sense | AAAGGATCCATGGCCGAAGCGCCCTCTCGA |
| mFthl17c-R |  | Reverse | AAAGATATCTTAGGACAAGCTGAGCTTGTCAAAGA |
